# Revision of *Massylaea* Möllendorff, 1898 (Stylommatophora, Helicidae)

**DOI:** 10.3897/zookeys.694.15001

**Published:** 2017-08-29

**Authors:** Houria Bouaziz-Yahiatene, Beat Pfarrer, Ferroudja Medjdoub-Bensaad, Eike Neubert

**Affiliations:** 1 Laboratoire de Production, sauvegarde des espèces menacées et des récoltes. Influence des variations climatiques. Département de Biologie. Faculté des sciences Biologiques et des Sciences Agronomiques. Université Mouloud Mammeri de Tizi-Ouzou. 15000. Algérie; 2 Natural History Museum of the Burgergemeinde Bern, Bernastr. 15, CH-3005 Berne, Switzerland

**Keywords:** *Massylaea*, *Eobania*, Algeria, Kabylie, revision, *Massylaea*, *Eobania*, Algérie, Kabylie, révision systématique

## Abstract

In this paper some helicoid species from eastern Algeria are investigated using a morphological and molecular approach. The investigation of the genital organs of *M. massylaea* (Morelet, 1851), the type species of the genus *Massylaea* Möllendorff, 1898, showed the same autapomorphic character states as are considered typical for *Eobania* P. Hesse, 1913. These findings are fully supported by the genetic analysis using two mitochondrial and three nuclear markers. Thus, the latter genus has to be considered a synonym of the former. Currently, three species are known to comprise the genus, viz. *M. massylaea*, *M. constantina* (E. Forbes, 1838), and *M. vermiculata* (O. F. Müller, 1774). Several nominal taxa from northern Africa are synonymised with one of the species mentioned here under *Massylaea*. The generic position of the so-called “*Massylaea*” species from the High Atlas Mountains in southern Morocco remains unresolved.

## Abstract (fr)

Dans cet article, certaines espèces d’Helicidae de l’est de l’Algérie sont étudiées par une approche morphologique et moléculaire. L’étude des organes génitaux de *M. massylaea* (Morelet, 1851), espèce de type du genre *Massylaea* Möllendorff, 1898, a montré les mêmes caractères autapomorphiques que ceux considérés comme typiques pour *Eobania* P. Hesse, 1913. Ces résultats sont pleinement soutenus par l’analyse génétique utilisant deux marqueurs mitochondriaux et trois nucléaires. Ainsi, ce dernier genre doit être considéré comme un synonyme de l’ancien. Actuellement, trois espèces sont connues pour comprendre le genre, à savoir *M. massylaea*, *M. constantina* (E. Forbes, 1838) et *M. vermiculata* (O. F. Müller, 1774). Plusieurs taxons nominaux de l’Afrique du Nord sont synonymes avec l’une des espèces mentionnées ici sous *Massylaea*. La position générique des espèces dites «*Massylaea*» des montagnes du Haut Atlas dans le sud du Maroc reste non résolue.

## Introduction

The north-eastern part of Algeria, which is called “La Grand Kabylie”, is an area seldom in the focus of malacological research. After the main active period in the second half of the 19^th^ century, which culminated in the monumental work of Bourguignat (1863-1864) on Algeria as a whole, and the very detailed list for the Kabylie published by Letournex (1870). Additional information was then supplied by Péchaud (1880), while the activities of Paul Pallary, who dominated the research on Northafrican molluscs after the death of Bourguignat in 1892 focused mainly on north-west Algeria and Morocco. It is the aim of the senior author of this paper to re-activate the malacological research in the area. Consequently, a combination of freshly collected specimens and a reworking of historical collections were chosen to approach this goal. On the long run, the establishment of a modern checklist for the area summarising and implementing the results of the latest research is planned.

Kobelt (1887) listed his findings of *Massylaea* in Algeria under the generalised genus *Helix* as was the use in these times. Later, Möllendorff (1898) established the genus *Massylaea* including the species *Helix massylaea* Morelet, 1851 and *Helix punica* Morelet, 1851 in this new group. Kobelt (1904: 34, etc.) in his biogeographical analysis used this new name for these two species, but erroneously related them with species from the Greek helicoid genus *Codringtonia* Kobelt, 1898 because of a superficially similar shell morphology. However, he also indicated (1904: 100) that particularly *H. punica* could also be considered as an aberrant form of *Alabastrina* Kobelt, 1904. This statement illustrates the uncertainty of how to define the genus, and which species to allocate there. Hesse (1911: 104) cites Pallary, who suggested to place *Helix bailloni* Kobelt, 1888 [from between Tiut und Mograr (= SW Algeria close to the Moroccan border) also under *Massylaea* and adds that this species was also found close to Constantine, the latter being a confusion with the true *M. massylaea*. Latest in 1915, Pallary started to use the name *Massylaea* also for the large and flat helicoid species living in the High Atlas of Morocco southwest of Marrakesch, like for example *Helix rerayana* Mousson, 1873, a use that pertains until today. In his anatomical work, Hesse (1915) restricted the use of *Massylaea* to species from east Algeria, but included the Westalgerian *Helix soluta* Terver, 1839 [currently *Alabastrina soluta*], although he found considerable differences even in the outer morphology of the genital organs. In the same work, Hesse also investigated *Eobania*, a generic name he had introduced earlier (1913: 13), and which is based on *Helix vermiculata* O. F. Müller, 1774. Interestingly, he already concluded that *Helix constantina* E. Forbes, 1838 belongs to his genus *Eobania*. However, he never noticed the high morphological similarity between *Massylaea* and *Eobania*. Schileyko (2006) listed *Massylaea* without further comment as a group near Helix Linnaeus, 1758, with 2-3 species from Tunisia, Algeria and Morocco.

Based on specimens collected by the first author of this paper and supplemented by museum’s specimens, a new try to disentangle the unclear taxonomic situation is taken using traits derived from shell morphology, anatomy of the genital organs as well as the results of an analysis of partial sequences of the genes COI, 16S, H3, 28S and ITS2.

## Material and methods

Living specimens as well as empty shells were collected in the Kabylie (eastern Algeria) during the last two years. For subsequent anatomical and molecular analysis, specimens were preserved and stored in 80% ethanol until dissection and DNA extraction. Specimens used in this study (both shells and preserved animals) are housed in the voucher collection of the first author and in the wet collection of the Natural History Museum of the Burgergemeinde Bern. Some of the sequences used in this study were downloaded from GenBank (https://www.ncbi.nlm.nih.gov).

First assessments of the shell morphological characters were done by using simple magnifying glasses. Preserved animals were dissected under LEICA M212 stereo microscope using thin tweezers. The genital organs of the specimens were removed from the body, the situs and further morphological details were investigated. After that, shells, genital situs and later details of the genital organs were photographed with a LEICA DFC 425 camera combined with a LEICA M205 C. The multifocal images were processed by using an imaging software (Imagic Switzerland).

### Molecular study

#### 1.1. Sampling

The specimens from Algeria used in this study were all collected by H. Bouaziz. Species from outside Algeria originate from the collections of Hutterer, Jochum, and Neubert. Data on sampling sites, voucher numbers, GenBank acession numbers and the identification of the specimens used are compiled in the Table 1.

**Table 1.** Species data.

Family	Species	Locality	Longitude	latitude	Voucher	GenBank acession number CO1	GenBank acession number 16S	GenBank acession number H3	GenBank acession number 28S	GenBank accession number 5.8S-ITS2-28S
Helicidae	Allognathus (Iberellus) hispanicus hispanicus	Ma 10 km 26 road. Escorca, Mallorca, 31SDE8608			EHUMC-1053	KM592543	KM592633			KM592718
Helicidae	*Eobania vermiculata*	Makouda, Tizi Ouzou/ Kabylie, DZ	36.7909	4.0659	NMBE 540544	MF564159	MF564112	MF564174	MF564128	MF564144
Helicidae	*Eobania vermiculata*	Beach between Agia Napa and Capo Greco, CY	34.9728	34.0427	NMBE 549959	MF564160	MF564113	MF564175	MF564129	MF564145
Helicidae	*Eobania vermiculata*	Kusadasi/ Izmir, TR	37.86	27.26	NMBE 549961	MF564161	MF564114	MF564176	MF564130	MF564146
Helicidae	*Helix melanostoma*	Kasserine, TN	35.1722	8.8307	NMBE 520822		MF564115	MF564177	MF564131	MF564147
Helicidae	*Helix melanostoma*	between Rabieux and Saint-Félix-de-Lodez/ Herault, F	43.6628	3.4409	NMBE 540550	MF564162	MF564116	MF564178	MF564132	MF564148
Helicidae	*Helix vladika*	Mokro close to Savnik, MNE	42.95	19.08	NMBE 23348	MF564163	MF564117	MF564179	MF564133	MF564149
Helicidae	*Hemicycla bidentalis*	Anaya, Tenerife, Canary Islands			MVHN-2160	KM592619	KJ458528			KJ458615
Helicidae	*Massylaea constantina*	Draâ-Ben Khedda/ Tizi Ouzou/ Kabylie, DZ	36.7318	3.9654	NMBE 534211a	MF564164	MF564118	MF564181	MF564134	MF564150
Helicidae	*Massylaea constantina*	Draâ-Ben Khedda/ Tizi Ouzou/ Kabylie, DZ	36.7318	3.9654	NMBE 534211b	MF564165	MF564119	MF564182	MF564135	MF564151
Helicidae	*Massylaea constantina*	Azaghar d’Ait Bouaddou, Bounouh, Tizi Ouzou, DZ	36.5214	3.9425	NMBE 540542	MF564166	MF564120	MF564183	MF564136	MF564152
Helicidae	*Massylaea constantina*	Makouda, Tizi Ouzou/ Kabylie, DZ	36.7909	4.0659	NMBE 540543	MF564167	MF564121	MF564184	MF564137	MF564153
Helicidae	*Massylaea constantina*	Ighil Bourmi, DZ	36.4872	4.0613	NMBE 540545	MF564168	MF564122	MF564185	MF564138	MF564154
Helicidae	*Massylaea massylaea*	Aurès Mountains/ Batna/ Kenchela, DZ			NMBE 519961	MF564169	MF564123	MF564180	MF564139	
Helicidae	*Otala punctata*	Tlemcen, DZ			MVHN-2186	KM592621	KJ458545			KJ458628
Helicidae	*Otala punctata*	Makouda, Tizi Ouzou/ Kabylie, DZ	36.7909	4.0659	NMBE 534228a	MF564170	MF564124	MF564186	MF564140	MF564155
Helicidae	*Otala punctata*	Makouda, Tizi Ouzou/ Kabylie, DZ	36.7909	4.0659	NMBE 534228b	MF564171	MF564125	MF564187	MF564141	MF564156
Helicidae	*Theba subdentata subdentata*	West of Aoulouz/ Souss-Massa-Draa, MA	30.7094	-8.2683	NMBE 549949	MF564172	MF564126	MF564188	MF564142	MF564157
Helicidae	*Tingitana “decussata*”	Montes de Kebdana, Djebel Sebaa Reyal/ Rif, MA	35.0297	-2.6134	NMBE 549840	MF564173	MF564127	MF564189	MF564143	MF564158

#### 1.2 DNA extraction, PCR amplification and sequencing

Total genomic DNA was extracted from the foot muscle tissue using Qiagen Blood and Tissue Kit (Qiagen cat nr. 69506) in combination with an QIAcube extraction robot (Protocol 430, DNeasy Blood Tissue and Rodent tails Standard). For this work we decided to use the following markers: Two mitochondrial gene fragments, Cytochrome c oxidase subunit I (COI) of 710 bp length and the 16S ribosomal RNA subunit (16S rRNA) for an approximately 480 base-pair segment. Three nuclear genes: the RNA (rRNA) cluster 5.8S-ITS2-28S of approx. 900 bp length (5.8S and 28S only partial, complete internal transcribed spacer 2), 28S ribosomal RNA partial sequence and the Histone 3 (H3) fragment. Primer pairs used in the PCR and sequencing are listed in Table 2.

**Table 2.** list of primer pairs used in PCR and sequencing.

Gene	Primer	Sequence	Reference
CO1	LCO1490	5’-GGTCAACAAATCATAAAGATATTGG-3’	Folmer et al. 1994
	HCO2198	5’-TAAACTTCAGGGTGACCAAAAAATCA-3’	
16S	16sF	5’-CGGCCGCCTGTTTATCAAAAACAT-3’	Palumbi et al. 1991
	16sR	5’-GGAGCTCCGGTTTGAACTCAGATC-3’	
28S	LSU-2	5’-GGGTTGTTTGGGAATGCAGC-3’	Wade and Mordan 2000
	LSU-4	5’-GTTAGACTCCTTGGTCCGTC-3’	
5.8S-ITS2-28S	LSU-1	5’-CTAGCTGCGAGAATTAATGTGA-3’	Wade and Mordan 2000
	LSU-3	5’-ACTTTCCCTCACGGTACTTTG-3’	
5.8S-ITS2-28S	ITS2ModA	5’-GCTTGCGGAGAATTAATGTGAA-3’	This work
	ITS2ModB	5’-GGTACCTTGTTCGCTATCGGA-3’	
H3	H3AD	5’-ATGGCTCGTACCAAGCAGACVGC-3’	Colgan et al.2013
	H3BD	5’-ATATCCTTRGGCATRATRGTGAC-3’	

PCR mixtures consisted of 12.5 µl of GoTaq G2 HotStart Green Master Mix (Promega M7423), 6.5 µl nuclease free H2O (Sigma-Aldrich, W4502), 1 µl of each primer and 2µl template DNA. The 25µl vol. mixtures passed through following listed reaction conditions. For COI, the cycling protocol begins with 3min at 95°C, followed by 35cycles of 1min at 95°C, 1min at 40°C and 1min at 72°C and finally, 5min at 72°C. For 16S the amplification conditions were 3min at 95°C, followed 35 cycles of 1min at 95°C, 1min at 50°C and 1min at 72°C, and finally, 5min at 72°C. ITS-2 and 28S shared the same cycle conditions: 1min at 96°C, followed 35 cycles of 30sec at 94°C, 30sec at 55°C and 1min at 72°C, and finally, 10min at 72°C. For H3, 3min at 95°C, followed 45 cycles of 45sec at 95°C, 45sec at 50°C and 45sec at 72°C, and finally, 10min at 72°C. The PCR condition for the new primer pair ITS2ModA and ITS2ModB are virtually the same as for the LSU-1/3 and varies only in the annealing temperature of 43°C.

The PCR product purification and sequencing was performed by LGC (LGC Genomics Berlin) and difficult/delicate sequences were sent for single tube sequencing to Microsynth (Microsynth Balgach Switzerland).

#### 1.3 Phylogenetic analyses

Geneious Ver.9.1.8 (Biomatters Ltd.) was used for Sequence processing and editing. MAFFT v.7.222 plugin of Geneious (Katoh and Standley 2013), was used with the default setting and the automated algorithm search setting. We decided defining the 16S fragment, ITS2 and 28S as single data blocks. The protein coding gene CO1 and H3 fragments were defined each in 2 data blocks: the first two codon positions as one block and the third codon position as a second. Partitionfinder Ver. 2.1 (Lanfear et al. 2012) was implemented to search the optimal evolutionary models for the partitions using the corrected Akaike Information Criterion (AICc). From the resulting evolutionary models, GTR +G was chosen for further Maximum Likelihood (ML) analysis. ML inference was computed with RAxML (Stamatakis, 2006), using Geneious ‘s plugin with the rapid bootstrapping setting, the search for the best scoring ML tree and 1000 bootstrapping replicates.

Bayesian Inference (BI) was performed using Mr Bayes v3.2.2 x64 (Huelsenbeck and Ronquist 2001; Ronquist and Huelsenbeck 2003; Ronquist et al. 2012) calculated through the UBELIx (http://www.id.unibe.ch/hpc) the HPC cluster at the University of Bern. The nucleotide model was set to 4by4 and a mixed evolution model with G+I rates was chosen, considering this to be the model best suited for the data of the concatenated sequences of 5 different genes (CO1, 16S, H3, ITS2, 28S). The Monte Carlo Markov Chain (MCMC) parameter was set as follow: starting with four chains and four separate runs for 20 × 106 generations with a tree sampling frequency of 1000 and a burn in of 25%. Trees were displayed on FigTree v1.4.3 (Rambaut 2012).

Abbreviations of shell measurements: D: shell diameter; H: shell height; PD: peristome diameter; PH: peristome height; W: number of whorls.

#### Abbreviations of collections used

**EHUMC** Euskal Herriko Unibersitatea Malacological Collection

**MHNG-MOLL** Muséum d’Histoire Naturelle Genève, collection Bourguignat, Switzerland

**MHNL** Musée des Confluences, Lyon, France

**MNHN** Muséum National d’Histoire Naturelle, Paris, France

**MVHN** Museo Valenciano de Historia Natural

**NMBE** Natural History Museum of the Burgergemeinde Bern, Switzerland

**NMSZ** National Museums of Scotland, Edinburgh

**SMF** Senckenberg Research Institut, Frankfurt am Main, Germany

## Results

### Molecular study

**Figure 1.**
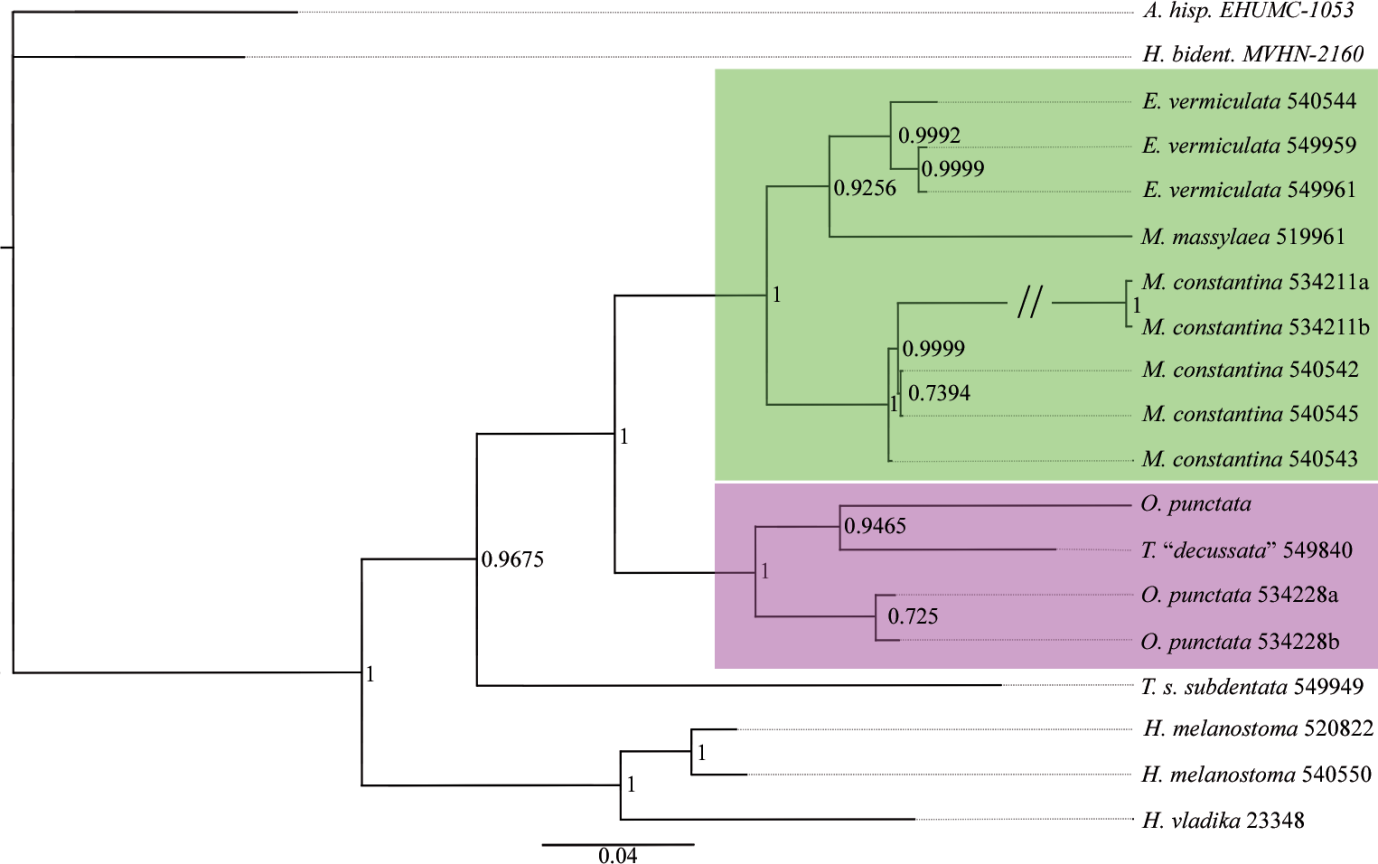
Bayesian Inference tree based on concatenated set of sequences (CO1 16S, H3, 28S, ITS2). Number on the nodes refer to posterior probabilities provided by the BI analysis.

**Figure 2.**
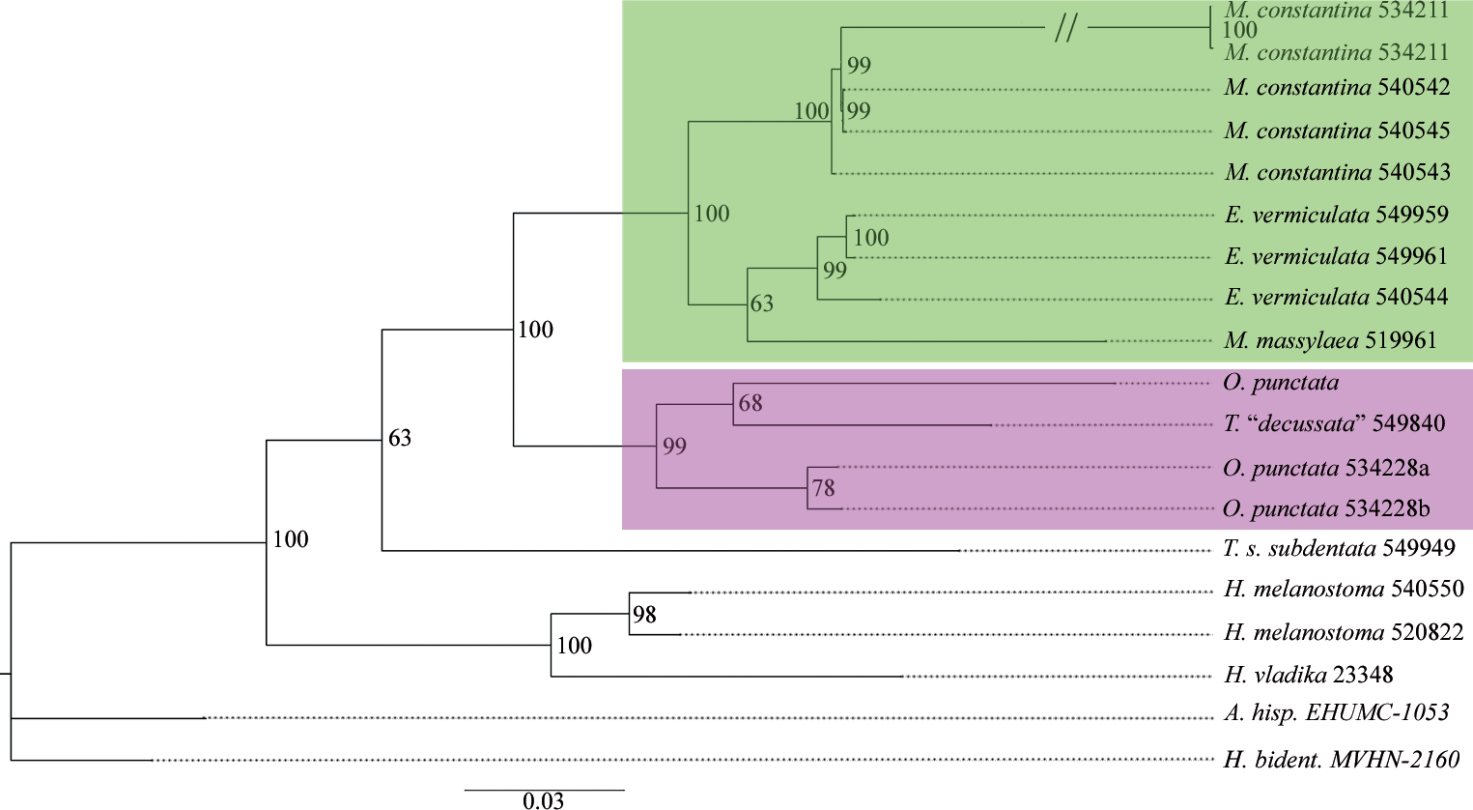
Maximum Likelihood (RAxML) tree based on the concatenated dataset (CO1 16S, H3, 28S, ITS2) Number on the nodes represent bootstrap support values from the ML analysis.

The p-distances of all markers of different *Massylaea* taxa are supplied as electronic supplementary files. The BI and RAxML analyses of the concatenated data set recovered the genus *Massylaea* and separated it from the *Otala*-clade with a maximal statistical support (Figs 1, 2).

Within *Massylaea*, the separation of species was relatively well supported. Although originating from Algeria, Cyprus and northwestern Turkey, the genetic difference between *vermiculata* populations was very low. In the Bayesian analysis (Fig. 1), the *constantina*-clade, however, detected an irregularity: the node that differentiated NMBE 540542 from NMBE 540545 showed only a low support, although both populations are only separated by a distance of 20 km. In the ML tree (Fig. 2), however, the same branching point was highly supported. In both trees, the position of *M. massylaea* within the clade is beyond any doubt.

Similar problems occurred in the *Otala*-clade. The specimens investigated included a species that is here identified as “*Tingitana decussata* Pallary, 1936”, which clustered within a group of *punctata*. In both trees, the two *punctata*-specimens NMBE 534228a and b had a low support, although they originate from the same population in eastern Algeria.

### Taxonomic implications

#### Genus Massylaea

Taxon classificationAnimaliaStylommatophoraHelicidae

Möllendorff, 1898

Massylaea Möllendorff, 1908; Nachrichtsblatt der Deutschen Malakozoologischen Gesellschaft 30 (9/10): 120.
Vermiculatiana Caziot, 1908; Mémoires de la Société Zoologique de France 20 (4): 439. Type species (by monotypy): Helix vermiculata O.F. Müller, 1774.
Eobania Hesse, 1913; Nachrichtsblatt der Deutschen Malakozoologischen Gesellschaft 45(1): 13. Type species (by monotypy): Helix vermiculata O.F. Müller, 1774.

##### Type species.

*Helix massylaea* Morelet, 1851 by tautonymy.

##### Diagnosis.

Large shells, spire flat to considerably raised, with or without a malleate surface structure, aperture without or only with a small labial ridge; penial chamber with a solid “false” penial papilla, epiphallus entering the penial chamber through a laterally situated pore, glandulae mucosae with many subdivided tubules, diverticulum very long.

#### Massylaea massylaea

Taxon classificationAnimaliaStylommatophoraHelicidae

(Morelet, 1851)

Figs 3 , 4–8

Helix massylaea Morelet, 1851; Journal de Conchyliologie 2: 354, pl. 9, fig. 1, 2 [La province de Constantine].
Helix punica Morelet, 1851; Journal de Conchyliologie 2: 352, pl. 9, fig. 3, 4 [habite la grande plaine de Temlouk, au sud-est de Constantine].
Helix massylaea var. concolor Bourguignat, 1863; Malacologie de l’Algérie I: 109, plate 9 fig. 9 [no type locality given].
Helix massylaea var. *conoïdea* Bourguignat, 1863; Malacologie de l’Algérie I: 109 [Ouled-Sultan (Deshayes)].
Helix nitefacta Bourguignat in Péchaud 1883; Excursions malagologiques dans le nord de l’Afrique de La Calle a Alger, d’Alger a Tanger: 99 [l’Aurès oriental à Aïn-Tamagra, au sud de Khenchala].
Helix massylaea var. zenatia Kobelt, 1887; Iconographie, (2) 3(1): 3 [Wed Zenati].
Helix punica var. speculatorum Kobelt, 1887; Iconographie, (2) 3(1): 6, Taf. 63, fig. 320–322 [El Kantara].
Massylaea (?) *severinae* Pallary, 1918; Bulletin de la Société d’histoire naturelle d’Afrique du Nord 9(7): 148 [Aïn el Bey (Constantine) (Philippe Thomas)].

##### Type specimens.

*massylaea*: 2 syntypes NHMUK 1893.2.4.43.5-6; *concolor*: syntypes MHNG-MOLL 118330/3 (Constantine on label in coll.); *conoidea*: not found in coll. Bourguignat; *nitefacta*: syntype MHNG-MOLL 118331/1; *zenatia*: not researched; *speculatorum*: not researched; *punica*: 3 syntypes NHMUK 1893.2.4.1240-1242; *severinae*: no type specimens found so far.

##### Other records.

Sigus, 36.1202°N 6.7849°E (Hesse 1920: 41); Tebessa, 35.4142°N 8.1010°E (Hesse 1920: 43, sub *punica*).

##### Diagnosis.

large grey-yellowish shells with maximum four brown spiral bands, aperture whitish to reddish brown, strong surface sculpture of longitudinal grooves.

##### Description.

Shell large, spire depressed to slightly broad conical, basic colour cream grey-yellowish with brown spiral bands; protoconch large (diameter ca. 5 mm), white; teleoconch whorls regularly increasing, with the last whorl considerably expanding before the aperture, rapidly declining at the aperture; suture deep, surface of teleoconch rough, covered by longitudinal, spirally arranged grooves, sometimes intersected by growth riblets and thus producing a pattern of longitudinal rectangles; spiral bands may be fully developed with maximum four spirals, but all variations including complete fusion of spirals may occur; aperture whitish to reddish brown with a thick lip, columellar part of aperture seldom with a ridge; peristome slightly thickened, umbilicus completely covered by a large reflection of the columellar part of aperture.

**Figure 3.**
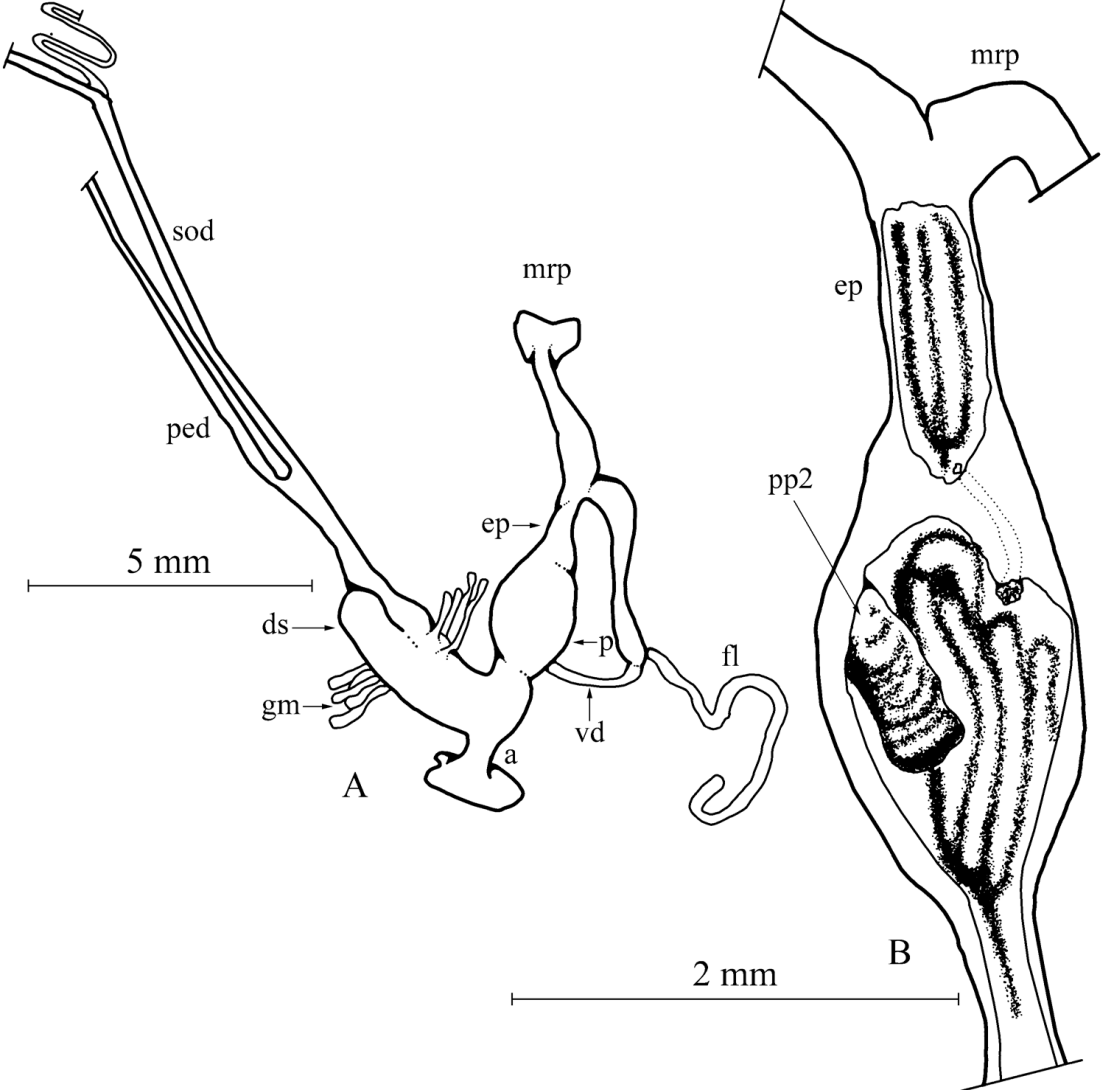
*Massylaea massylaea*, morphology of the genital organs; NMBE 519961/1, Aurés Mountains (sequenced specimen). **A** situs of the hermaphroditic genitals **B** penis opened to show internal structure.

Genital organs (only the single subadult specimen (NMBE 519961, sequenced specimen) could be investigated, Fig. 3): Penis short, bulbiform; epiphallus reaching twice the length of penis, with the penis retractor muscle inserting in the distal third of epiphallus; tubiform flagellum reaching the length of penis + epiphallus; penial lumen filled with longitudinal fleshy pilasters; penial chamber with a solid penial papilla (pp2, see also Neubert and Bank 2006); epiphallus opening into the penial chamber via a small pore opposite the “false papilla”.

Female part almost undeveloped, the glandulae mucosae only represented by a few small tubules; all other female structures only weakly developed.

Measurements: Syntype *massylaea*: H = 28.4 mm; D = 40.1 mm; PH = 12.6 mm; PD = 20.5 mm; W = 4.75.

##### Distribution.

Kobelt (1887) supplied data on the distribution of both, *massylaea* and *punica*, and stated that they may occur in hundreds of specimens in a single locality. Given the fact that we here consider *punica* a synonym of *massylaea*, this taxon turns out to be one of the most widespread helicoid species in the southern part of the Eastalgerian mountain range covering southwestern parts of the province of Constantine, and parts of the provinces of Biskra and Blida westwards to Schott el Hodna.

Remarks: The variation in shell morphology mainly concerns the elevation of the spire, which may be rather flat to considerably raised. The second character state that varies is the formation, number and colour of the spiral bands. These may be reddish- to chestnut-brown, some may miss completely or in parts, or are fused to form a cloudy brownish surface. Although Kobelt (1887: 3) states that his specimens usually had spiral bands he separated one form without spiral bands under the name *zenati*, claiming that this form only occurs at this single locality, from which already Bourguignat named his var. concolor. According to the specimens known today, spiral banding is quite stable, but there are all variations seen from five bands to completely unicoloured shells.

Hesse (1920: 43, sub *punica*) reports that he received three specimens of this species from Tunisia “Redyef im südlichen Tunis [= Al Rudayyif, Gafsa]. This record has not been reconfirmed by modern collections and is probably based on a confusion with *vermiculata*.

**Figures 4–8.**
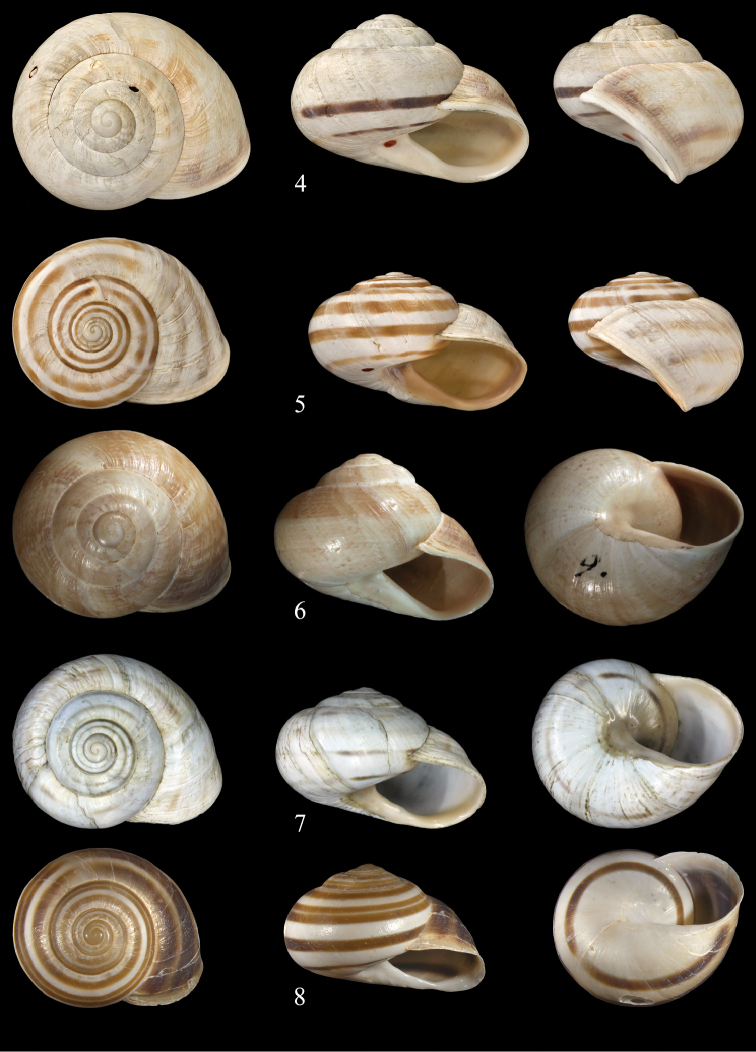
*Massylaea massylaea* (Morelet, 1851). **4** syntype *Helix massylaea* NHMUK 1893.2.4.43.5, D = 40.1 mm **5** syntype *Helix punica* NHMUK 1893.2.4.1240, D = 36.8 mm **6** syntype *concolor* MHNG-MOLL 118330, D = 36.9 mm **7** syntype *nitefacta* MHNG-MOLL 118331, D = 35.5 mm **8** NMBE 519961, Aurés Mountains, D = 33.65 mm (sequenced specimen). All figures Neubert, natural size.

#### Massylaea constantina

Taxon classificationAnimaliaStylommatophoraHelicidae

(E. Forbes, 1838)

Figs 9 , 10–12

Helix constantina E. Forbes, 1838; Annals of Natural History, II: 251, pl. XI, fig. 1 [In waste places among nettles at Bougia].
Helix cirtae Terver, 1839; Catalogue des Mollusques...: 11, pl. 1, fig. 1 [Bone].
Helix constantinae var. bifasciata Bourguignat, 1863; Malacologie de l’Algérie I: 114 [La Calle].
Helix constantinae var. conoidea Bourguignat, 1863; Malacologie de l’Algérie I: 114, plate 10 fig. 7 [Constantine].
Helix constantinae var. depressa Bourguignat, 1863; Malacologie de l’Algérie I: 114 [Ouled-Sultan].
Helix constantinae var. maxima Bourguignat, 1863; Malacologie de l’Algérie I: 114 [Constantine].
Helix constantinae var. minima Bourguignat, 1863; Malacologie de l’Algérie I: 114 [Bone].
Helix constantinae var. trifasciata Bourguignat, 1863; Malacologie de l’Algérie I: 114 [La Calle].

##### Type specimens.

*constantina*: no type specimens could be identified in the E. Forbes collection in NMSZ; *cirtae*: syntype MHNL 45001107; the type specimens for the varietal names of Bourguignat are not identifiable in his collection.

##### Additional specimens.

Ighil Bourmi, 36.4872 4.0613, 1297 m alt., 24.5.2015, Bouaziz, NMBE 540545/1, Makouda, Tizi Ouzou/ Kabylie, 36.7909 4.0659, 440 m alt., 22.3.2015, Bouaziz, NMBE 540543/3, Azaghar, Bounouh, Tizi Ouzou, 36.5214 3.9425, 432 m alt., 26.4.2015, Bouaziz, NMBE 540542/9, Draa Ben Khedaa/ Tizi Ouzou, 36.7318 3.9654, 50 m alt., 6.1.2015, Bouaziz, NMBE 534211/20.

##### Diagnosis.

Medium sized shell, teleoconch smooth, and aperture with a raised columellar ridge.

##### Description.

Shell medium sized, with a globular or broad conical spire, basic colour white to grey, always with up to five brown spiral bands; protoconch large (diameter ca. 4 mm), white; all teleoconch whorls regularly increasing, with the last whorl rapidly declining at the aperture; suture of moderate depth, surface of teleoconch smooth; usually with five spiral bands with spiral 2+3 often very close or almost merging; aperture always porcelain white with a thick lip, columellar part of aperture with a raised ridge; peristome slightly thickened, umbilicus completely covered by a large reflection of the columellar part of aperture.

**Figure 9.**
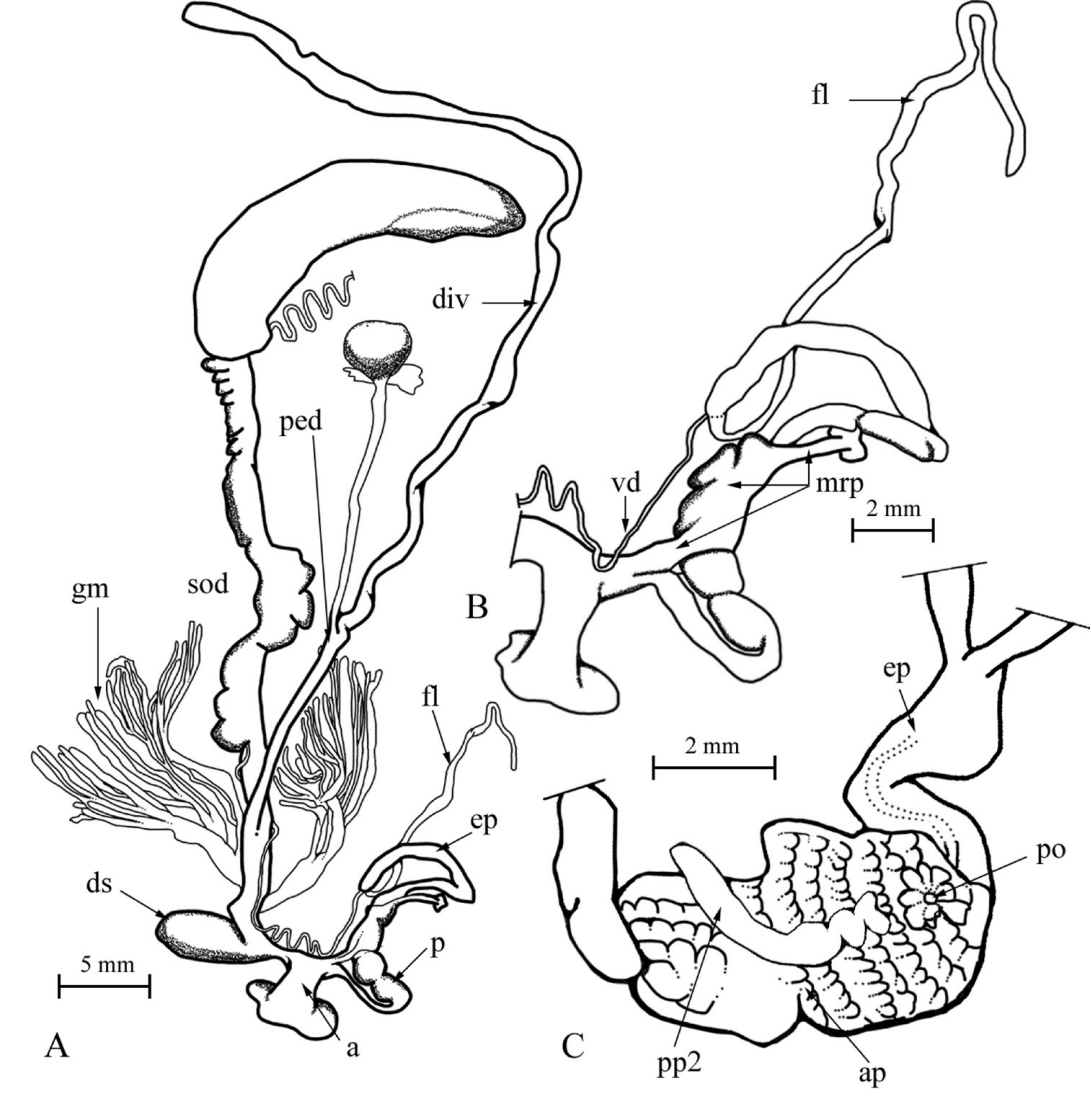
Genital organs of *M. constantina*; NMBE 540542, Azaghar; **A** situs **B** outer morphology of male organ **C** interior of penis. Abbreviations used: a = atrium; ap = annular pad; div = diverticulum; ds = dart sac; ep = epiphallus; fl = flagellum; gm = glandulae mucosae; mrp = penial retractor muscle; p = penis; ped = pedunculus; po = pore of penial papilla; pp2 = second penial papilla; sod = spermoviduct; vd = vas deferens.

Genital organs (Fig. 9A–C): penis subdivided in three parts, with an elongate distal tube connecting to the atrium, and a bilobed muscular proximal part with the internal boundary marked by an annular pad (Giusti et al. 1995); epiphallus longer than penis, enveloped by a strong penial retractor muscle that connects with a fascicle to the atrium, flagellum a long, simple tube; internally, the proximal penial chamber filled by a solid penial papilla (pp2, see also Neubert & Bank 2006); epiphallus opening into the penial chamber via a small pore.

Dart sac opening laterally into the short vagina; glandulae mucosae with two central stems giving rise to at least three subsequent branches with at least 40 tubules; diverticulum branches off in a central position from the pedunculus reaching a length of at least 30 mm.

Measurements: syntype *cirtae*: H = 21.3 mm; D = 27.2 mm; PH = 8.6 mm; PD = 12.9 mm; W = 4.75.

##### Distribution.

This species is known from Tizi Ouzou in the Grand Kabylie towards the northern parts of the province of Constantine. In many places it lives in sympatry with *M. vermiculata*.

##### Remarks.

This species proved to be quite stable in terms of conchological traits. The number of five spiral bands is very stable as well as the white and smooth teleoconch. Some colour morphs of *M. vermiculata* look quite similar; however, so far all shells of the latter species could be differentiated by presence of the malleate teleoconch surface. This surface structure may be reduced to a small area above and around the aperture, but it is always clearly discernible. It differs from *M. massylaea* by its considerably smaller shell, the high globular spire, and the smooth teleoconch surface.

**Figures 10–12.**
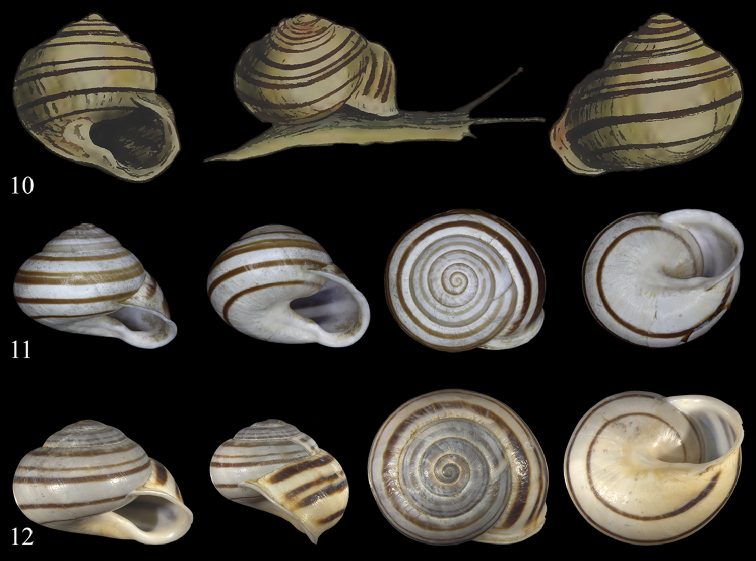
*Massylaea constantina* (E. Forbes, 1838). **10** Original figure of E. Forbes, pl. XI, Figure 1 [1839], size adopted to next figure **11** syntype *cirtae* MHNL 45001107, D = 27.2 mm **12** NMBE 534211, Draa Ben Khedaa, D = 30.25 mm. All figures Neubert/Bochud, natural size.

#### Massylaea vermiculata

Taxon classificationAnimaliaStylommatophoraHelicidae

(O. F. Müller, 1774)

Figs 13–16

Helix vermiculata O. F. Müller, 1774; Vermium terrestrium et fluviatilium 2: 21 [In Italia sabulosis juxta torrentes].
Helix bonduelliana Bourguignat, 1863; Mollusques nouveaux, litigieux ou peu connus, fasc. 1: 9, plate 3 figs 1–4 [Province d’Oran].
Helix vermiculata var. albida Bourguignat, 1863; Malacologie de l’Algérie I: 112, plate 8 fig. 10 [La Calle].
Helix vermiculata var. aspersa Bourguignat, 1863; Malacologie de l’Algérie I: 112 [Cherchell].
Helix vermiculata var. expallescens Bourguignat, 1863; Malacologie de l’Algérie I: 112 [Environs d’Alger, Blidah].
Helix vermiculata var. minuta Bourguignat, 1863; Malacologie de l’Algérie I: 112 [Ile de Galite].
Helix vermiculata var. trizonata Bourguignat, 1863; Malacologie de l’Algérie I: 112 [Philippeville].
Helix fleurati Bourguignat, 1868; Histoire Malacologique de la Régence de Tunis: 12, plate 1 fig. 1–3 [Env. de Tunis (Champs au sud et au sudest de Tunis, entre un vieux puits espagnol et les collines de Sidi ben Hassen et de la forteresse Bordj el Raïs. Ruines d’Oudena. Ruines d’Utique et de Carthage. non loin de la chapelle Saint-Louis]
Helix fleurati var. obesa Bourguignat, 1868; Histoire Malacologique de la Régence de Tunis: 13 [no locality information given].
Helix fleurati var. subcarinata Bourguignat, 1868; Histoire Malacologique de la Régence de Tunis: 13, plate 1 fig. 4 [no locality information given].
Helix (Macularia) vermiculata var. conoidea Issel, 1880; Annali del Mus. Civ. di St. Nat. di Genova, Vol. XV: 263 [Sahel, fra Susa e Bir el Buita e fra Susa ed El Gem].
Helix (Macularia) vermiculata var. depressa Issel, 1880; Annali del Mus. Civ. di St. Nat. di Genova, Vol. XV: 263 [Cartagine].
Helix (Macularia) vermiculata var. minuta Issel, 1880; Annali del Mus. Civ. di St. Nat. di Genova, Vol. XV: 264 [Is. Galita, Galitone, Aguglia, Gallina (Violante, 1877). Cartagine (Bellucci, 1875)].
Helix toukriana Bourguignat in Péchaud 1883; Excursions malagologiques dans le nord de l’Afrique de La Calle a Alger, d’Alger a Tanger: 37 [hauts plateaux du Sersou, entre Aïn-Toukria et le Nahr-Ouassel, dans la direction de Sebaïn-Aïoun].
Helix aecouria Letourneux et Bourguignat, 1887; Prodrome de la malacologie terrestre et fluviatile de la Tunisie: 7 [Environs d’Houmt-Souk dans l’ile de Djerba].
Helix vermiculata var. saharica Kobelt, 1887; Iconographie, (2) 3(1): 9, Taf. 6, fig. 343–345 [Biskra].

##### Type specimens.

*bonduelliana*: 1 syntype MHNG-MOLL 118415; *aecouria*: 3 syntypes MHNG-MOLL 118413; *fleurati*: syntypes MHNG-MOLL 118440/7 (Env. de Tunis); *toukriana*: syntype MHNG-MOLL 118487; *saharica*: not researched.

##### Diagnosis.

Medium sized shell, teleoconch with a malleate surface sculpture, and aperture with a slightly raised columellar ridge.

##### Description.

shell medium sized, with a globular to depressed conical spire, basic colour white to grey, up to five brown spiral bands may be present or completely missing; protoconch large (diameter ca. 4 mm), corneous to white; whorls regularly increasing, the last whorl declining at the aperture; teleoconch suture of moderate depth, surface of teleoconch with a characteristic malleate sculpture (sometimes only present close to the aperture!); spiral bands 2+3 often merging, and bands may fuse to a large brown area on the last whorl before the aperture; aperture usually porcelain white with a thick lip, columellar part of aperture with a raised ridge; peristome slightly thickened, umbilicus completely covered by a large reflection of the columellar part of aperture.

Genital organs (after Giusti et al. 1995; Neubert and Bank 2006; Holyoak and Holyoak 2017): penis clubshaped, bipartite; the bilobed muscular proximal part not visible in outer morphology; epiphallus longer than penis; penial retractor muscle simple, attaching at the boundary of penis and epiphallus; internally, the proximal penial chamber filled by a solid penial papilla, epiphallus opening into the penial chamber via a small pore or on top of a flat papilla.

Dart sac opening laterally into the short vagina; glandulae mucosae with two central stems giving rise to at least three subsequent branches with at least 40 tubules; diverticulum branches off in a central position from the pedunculus surpassing the bursa copulatrix enormously.

##### Distribution.

This species is widely recorded throughout Tunisia and eastern Algeria.

##### Remarks.

The synonymy list and illustrations only cover synonyms of *M. vermiculata* important for the area from east Algeria and Tunisia. Here, this species inhabits Mediterranean shrublands as well as the wooded hinterland. It also tolerates coastal dunes with salty spray, and semiarid steppes. Holyoak and Holyoak (2017) justify the synoynmisation of *constantina* with *vermiculata* with the similar morphology of the genital organs and the wide overlap in shell size and banding pattern. However, in many land-snails, closely related species cannot be differentiated by the morphology of their genital organs. More attention should be paied to the construction of the penis (bilobed muscular proximal part visible from outside or not) and the variability of attaching system of the retractor muscle, which is much larger (and also connects to the atrium) in the specimen of *constantina* than in any *vermiculata* seen so far. The most important character state that separates the species is the absence of any malleation on the shell surface in *constantina*. Additionally, the phenotypic plasticity of the shells of *vermiculata* is markedly contrasted by the congeneric *M. constantina*, which is extremely stable in respect of the spiral banding pattern.

One species mentioned by Kobelt (1887) as a closely related species to his *vermiculata-constantina*-complex is *Helix bonduelliana* Bourguignat, 1863, with the type locality “Province d’Oran” in western Algeria. Kobelt doubts the correctness of this locality, and speculates that it might originate from Tunisia. According to his personal experience in Algeria, *M. vermiculata* reaches the Isser, but does not expand much to the west of this river. The only exception he found was Cherchell west of Algiers, where the species occurred in large numbers, but restricted to and around the harbour, so it can be considered being introduced there. It is not clear how the situation is today along the central and western Algerian coast, but Kobelt’s lines can be seen as an information on the natural range of this species in northern Africa. In the same year, Bourguignat (1887: 8) records *H. bonduelliana* from Ghardimaou (Tunisia) (= MHNG-BBT 118417/3). Bourguignat’s collection has another record from “environs de Tunis” under MHNG-MOLL 18416/1. Later, Pallary (1898) mentions his and Debeaux’s unsuccessful attempts to recollect the species in Oran. Summarising it can be said that the type locality of *H. bonduelliana* is apparently wrong, and the specimens are very probably of Tunisian offspring. This nominal taxon fully falls into the colour variation of *M. vermiculata*, which can reach from completely white shells as exemplified by *H. fleurati* (Fig. 15) to the typical morph as seen in *H. aecouria* (Fig. 14). The shell shape, however, is quite stable in most of these forms, and typical for *M. vermiculata*. The synonymisation of *saharica* Kobelt, 1887 needs reconfirmation by study of the type specimens.

**Figures 13–16.**
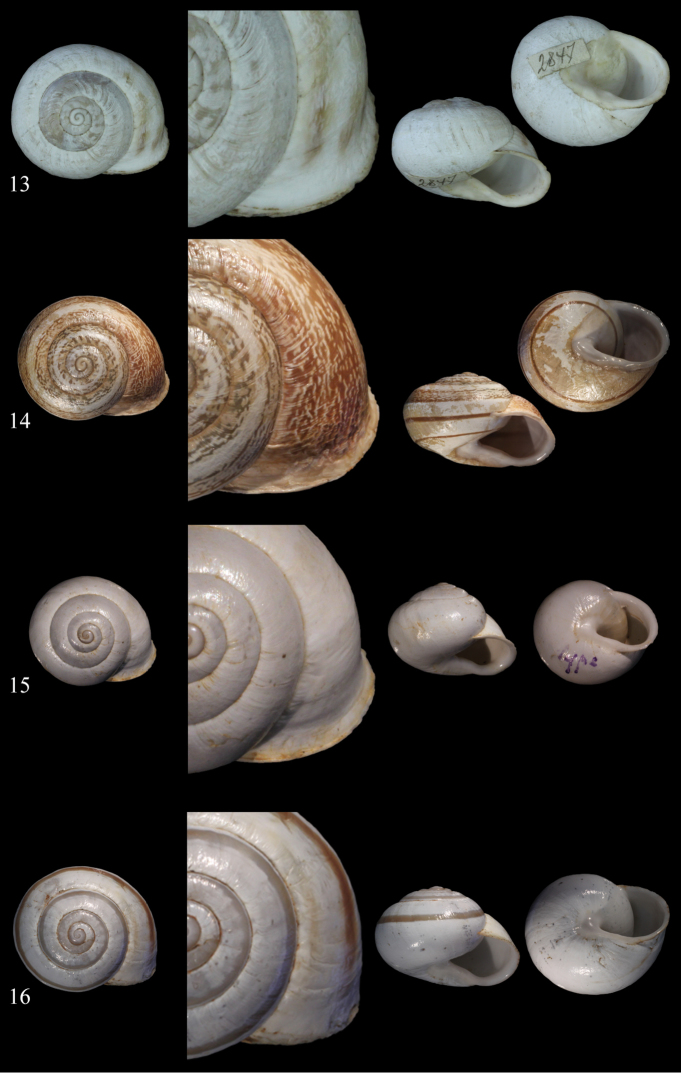
*Massylaea vermiculata* (O. F. Müller, 1774). **13** syntype *Helix toukriana* MHNG-MOLL 118487, D = 29.7 mm **14** syntype *Helix aecouria* syntype MHNG-MOLL 118413, D = 28.4 mm **15** syntype *Helix fleurati* MHNG-MOLL 118440, D = 23.9 mm **16** *Helix bonduelliana* Bourguignat, 1863 syntype MHNG-MOLL 118415, D = 27.0 mm. — All figures Neubert/Bochud, natural size.

## Discussion

The results of our study strongly support the monophyly of the genus *Massylaea*. This is evidenced by traits of the genital organs as well as by the genetic analysis, which is based on two mitochondrial and three nuclear markers.

Although only a subadult specimen of *M. massylaea* was available for investigation, the autapomorphic character states of presence of a “blind” penial papilla in combination with a separate epiphallial pore could clearly be detected (Fig. 3). This type of internal penial construction is also seen in *M. constantina* (Fig. 9) and in *M. vermiculata* (Neubert and Bank 2006). It differs profoundly from other helicoid genera like for example the syntopic *Otala punctata* (O. F. Müller, 1774), which shows the plesiomorphic type of a bipartite penis with two subsequent penial papillae (De Mattia and Mascia 2011).

In the genetic analysis, the *Massylaea*-clade is supported by high bootstrap values (Figs 1, 2). Within the clade, a separation of *M. constantina* from a combined cluster *vermiculata-massylaea* can be seen. The *constantina* population NMBE 534211 shows an enormous number of base-pair substitutions if compared to the other three congeners. The distance matrixes show that the generated 5.8S-ITS2-28S sequence is responsible for the caused deviation. In an ITS2 alignment, the NMBE 534211 population showed a higher variation, often up to 84bp, in the complete second Internal Transcriber Spacer region, in comparison to the congeners. NMBE 534211 from Draâ-Ben Khedda seems to be constituted by a faster evolving population, indicated by the much longer branch reflecting the higher base pair substitutions. We could hypothesize that we probably are witnessing the beginning of a speciation process, but we have to collect more specimen from the complete distribution area to be more conclusive about the existing species boundaries.

The generic name *Massylaea* Möllendorff, 1898 has been widely used for a number of species and thus cannot be treated as a nomen oblitum. It has precedence over *Eobania* Hesse, 1913, and the widespread species *Helix vermiculata* O. F. Müller, 1774, has to be classified under this generic name. Currently, the use of *Massylaea* should be restricted to the three species treated here; the so-called “*Massylaea*” species from the High Atlas Mountains in southern Morocco are widely unknown, and probably form a separate generic entity. At least 12 nominal taxa can be affiliated to this radiation, but species delimitation poses a major problem at the moment.

**Figure 17.**
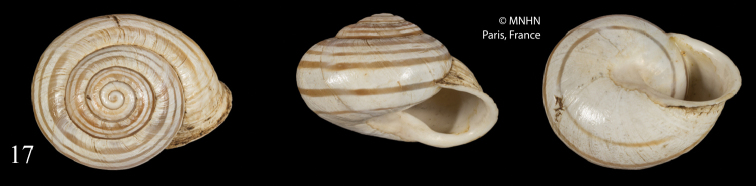
*Helix boghariensis* Debeaux, 1857 [Rochers en face le village arabe Ksar·el-Boghari]; syntype MNHN IM-2000-31714, D = 34.0 mm. Figure 17 by courtesy of MNHN, Paris, natural size.

Already Kobelt (1887) remarked the similarity of *Helix boghariensis* Debeaux, 1857 (syntype Fig. 17), with the *constantina*-*vermiculata*-group. In fact the shell of this species is close to *M. constantina*, and lacks the teleoconch sculpture of *M. vermiculata*. Kobelt remarked that the type locality Boghar is far from the range of *M. constantina*, which was true at his time. Due to the collections of the senior author we now know that *M. constantina* also inhabits the area of Tizi Ouzou. The distance (as the crow flies) between these localities is about 150 km. From a shell morphological point of view there is no evidence for any major difference between *constantina* and *boghariensis* supporting the separation of the latter as a species in its own rights. This question can only be sorted out by an investigation of animals from Boghar. Holyoak and Holyoak (2017) synonymise this taxon with *E. vermiculata* without further comments.

The enormous radiation of Helicid species and genera in northern Africa is poorly understood and still in a chaotic state, and often complained about like recently in Holyoak and Holyoak (2017). Although not in the centre of the helicoid radiation in northern Africa, our results may contribute to some clarifications like for example in the “*Otala*-clade”. By chance, a specimen of *T.* “*decussata*” (nomen nudum) could be included in our study. This taxon is conchologically similar to *Archelix minettei* Pallary, 1917 from “Tarzout-du-Guigou”, which is the type species of *Tingitana* Pallary, 1918, a genus used to accommodate a number of helicoid species from all over Morocco. The position of *T.* “*decussata*” in our concatenated trees (see Figs 1, 2) indicates that *Tingitana* is close to or even identical with *Otala*. This result coincides with Razkin et al. (2015), who found that *T. orientalis* Pallary, 1918 from Berkane clusters within the *Otala* clade. However, it is not clear whether this species is a *Tingitana* in its original sense, or rather a classical *Otala*. Although we have a more clear evidence for a synonymisation of *Tingitana* with *Otala* we still consider this synonymy as premature as long as evidence through study of anatomical and genetic data of the type species of the genera is supplied. It should be mentioned that the form “*decussata*” lives in the summit area of the Kebdana (leg. R. Hutterer); the population consists exclusively of strongly keeled specimens.

Holyoak and Holyoak (2017) revisited the problems within *Otala* and *Eobania* in northern Africa adding valuable distribution data. However, some details are astonishing. The authors cover the complete distribution area of the genus *Massylaea* and collect (and synonymise) a reasonable number of available species-level names, but completely omit *Helix massylaea* Morelet, 1851! The only reference to the genus is restricted to a note, where it is mentioned as a host of *Helix soluta* Michaud, 1833, perpetuating the erroneous ideas of Kobelt (see introduction). A concept for *Massylaea* is completely missing, and all taxa are lumped under *Eobania vermiculata*. Puzzlingly, the only exception is *Helix punica* (from south of Constantine), which is affiliated by the authors to *Loxana* Pallary, 1899. This genus is based on *Helix beaumieri* Mousson, 1873, which lives in the High Atlas south of Marrakech. It is currently considered to constitute a subgenus of *Alabastrina* Kobelt, 1904; the new concept of *Loxana* is not explained, delimited or justified. Under the same generic name *Loxana*, the enigmatic *Helix rerayana* Mousson, 1873 is treated, a species which originates from the same larger area as *H. beaumieri*, but differs enormously in shell shape, so a congeneric position for these two taxa will require good arguments (which are not supplied in this paper). All names allocated by us to the three accepted taxa under *Massylaea* (and scrutinized by checking and presenting the type specimens), are lumped by Holyoak and Holyoak (2017) under *E. vermiculata*, a concept, which does not comply with our genetic data.

Concluding it can be said that still, the chaos is not fully disentangled, and that the rigorous lumping of taxa is probably not fully supported. We agree with Holyoak and Holyoak (2017: 420) that “it has been taken as axiomatic that the species recognised should be identifiable from morphological characters, of shells, genital anatomy, or both”. But then, prior to any decision taken, the initial point should be the study and presentation of type specimens. This is a major service to other students of the fauna and greatly facilitates the understanding of subsequent decisions made. It could help to clarify the identity of species-level taxa used in genetic studies, and thus constitute a major contribution towards stabilisation of taxonomy and nomenclature.

## Supplementary Material

XML Treatment for Massylaea

XML Treatment for Massylaea massylaea

XML Treatment for Massylaea constantina

XML Treatment for Massylaea vermiculata
